# Validation and initial results of surveys exploring perspectives on risks and solutions for diagnostic and medication errors in primary care in Sweden

**DOI:** 10.1080/02813432.2020.1841531

**Published:** 2020-12-11

**Authors:** Rita Fernholm, Caroline Wachtler, Karolina Malm-Willadsen, Martin J. Holzmann, Axel C. Carlsson, Gunnar H. Nilsson, Karin Pukk Härenstam

**Affiliations:** a Division of Family Medicine and Primary Care, Department of Neurobiology, Care Sciences and Society, Karolinska Institutet, Huddinge, Sweden; b Department of Medicine, Stockholm, Sweden; c Functional Area of Emergency Medicine, Karolinska University Hospital, Stockholm, Sweden; d Department of Learning, Informatics, Management and Ethics, Medical Management Centre, Stockholm, Sweden

**Keywords:** Patient safety, primary health care, medical errors, continuity of patient care, risk assessment, quality of health care

## Abstract

**Objective:**

To (1) validate and (2) display initial results of surveys to health care professionals and patients on the importance and mitigation of specified risks for diagnostic and medication errors.

**Design:**

For validation, psychometric properties were analysed by assessment of construct validity and internal consistency by factor analysis. Non-parametric analyses were used concerning areas of risk, and top ranking of solutions were reported descriptively.

**Setting:**

Primary health care in Sweden.

**Participants:**

Health care professionals (HCPs); including physicians, nurses and practice managers, as well as patients who had experienced diagnostic or medication errors.

**Main outcome measures:**

Psychometric properties of the surveys. Median ratings for risks and top rankings of solutions for professionals and patients.

**Results:**

There were 939 respondents to the HCP survey. Construct validity resulted in a model with four dimensions: Patient-provider level; Support systems for every day clinical work; Shared information and cooperation between different caregivers; Risks in the environment. Internal consistency was acceptable with Cronbach’s α values above 0.7. Confirmatory factor analysis generally showed an acceptable fit. Initial results from the professionals showed the importance of continuity of care, a nationwide on-line medical platform and cooperation in transfer of care. The patient survey could not be validated because of low response rate.

**Conclusion:**

The HCP survey showed some contradicting results regarding model fit and may be tentatively acceptable but validity needs further study. HCP survey answers indicated that relational continuity of care and a nationwide on-line medical platform are highly valued.

**Current awareness**
- Health care professionals and patients are rather untapped sources of knowledge regarding patient safety in primary health care

**Main statements**
- Validation is performed on a new survey capturing rating of risks and solutions.
- The validation of the health care professional survey is tentatively acceptable.
- Survey answers indicate that health care professionals’ and patients’ perspectives are complementary.

## Introduction

Patient safety in primary health care (PHC) demands increased focus, because most health care encounters happen in PHC. Diagnostic error – delayed, missed, or incorrect diagnoses [1] – are common in PHC, especially in cases of serious preventable harm [1–3]. Medication errors are also common in PHC, occurring in 3–10% of all doctor visits where drugs are prescribed [4]. Few interventions for safer PHC have been tested and none implemented systematically [5]. Of tested interventions, not all have been successful: for example, attempting safer care through computer-based interpretation of atrial fibrillation led to diagnostic errors [6].

The LINNEUS collaboration identified four main areas of patient safety in PHC [7]: diagnostics, medication, communication and organisation. Patient involvement in safety work is critical [8], and it is well understood that intervention implementation is facilitated when solutions originate from people present in the system [9].

The Yorkshire Contributory Factors Framework (YCFF) is an evidence-based framework of factors contributing to patient safety incidents in hospital settings [10]. A qualitative study with patients and carers explored patient safety factors in primary care compared to YCFF [11]. Furthermore, General Practitioner (GP) perspectives on patient safety incidents have been explored [12]. However, it is still unknown what contributing factors or risks are considered important for mitigation from both health care professionals’ (HCPs’) and patients’ perspectives. Our group has earlier conducted a qualitative study to identify factors important for understanding risks and solutions for patient safety issues in PHC [13]. These results were used in the present study to guide the design of the surveys for HCPs and patients. There is an instrument for assessing patient safety culture in primary care (Manchester Patient Safety Framework) but no instrument for assessing which patient safety issues should be prioritised to address.

The aim of this study was to (1) validate and (2) display initial results of surveys to HCPs and patients on the importance and mitigation of specified risks for diagnostic and medication errors.

## Methods

### Study design

This is a validation study and initial report of surveys investigating how HCPs and patients rate specified risks and risk mitigation strategies.

### Survey construction

Two surveys were constructed, aimed at HCPs and patients respectively. Questions about risk were formulated as follows: ‘How big of a risk (or problem) do you believe “X” is?’. Respondents rated answers on a scale from 0 to 10, from ‘not at all’ to ‘very big’. Questions are displayed in the full surveys (Supplementary Appendices 1 and 2). Questions about risk mitigation used drag-and-drop alternatives with most preferred possible solution in the top position. The program Questback essentials® (fall edition 2018) was used to construct the online surveys.

A cognitive test of the questions, performed by sending the surveys to ten HCPs and two patients, led to minor linguistic adjustments.

### Participants and data collection

Data was collected during 2018 in Swedish PHC. Only GP practices were included, no nursing homes or home nursing care.

We recruited HCPs (practice managers, physicians and nurses) by sending e-mails to practice managers at randomly selected GP practices all over Sweden, asking for e-mail addresses to physicians and nurses. We received e-mail addresses to 3 556 HCPs (2 210 were doctors, 832 nurses, 514 practice managers). The practice managers were 93% doctors or nurses by training. All responses were electronic. This was convenience sampling with many participants and a broad geographic spread.

We recruited individuals who had themselves, or had a relative who had, experienced diagnostic (70%) or medication error (30%) in contact with PHC. Individuals were randomly chosen from Landstingens Ömsesidiga Försäkringsbolag (LÖF), a nationwide non-punitive malpractice carrier and insurance company, and had received compensation for preventable harm during 2010–2017. We contacted 426 individuals *via* e-mail if the address was known (86%), otherwise by paper post (14%). If the individual was a minor, parents were contacted. We excluded cases in which the patient had died. There was no relationship between patients and HCPs. Two reminders were sent to HCPs. One reminder was sent to patients.

Electronic data was anonymously collected and stored in the password protected program Questback. Paper data was anonymised and filed electronically in Questback and then stored safely, only available to researcher R.F. Data remained anonymised throughout the study.

### Survey validation

The HCP survey was validated using factor analysis to assess construct validity (assessment of relationships between items and relations between items and an underlying dimension) and internal consistency (assessment of whether items assumed to measure the same general construct produced similar scores). The validation was done according to the guidelines of Cabrera–Nguyen [14] and recommendations from Jackson [15]. When examining the survey data with respect to underlying assumptions for the factor analysis it was clear that some items had a non-normal distribution. Therefore, the factor analysis was performed on a polychoric correlation matrix and an estimation method was chosen that do not rely heavily on the normal assumption, see further details below. Bartlett's test of sphericity performed on the survey data gave a *p*-value <0.0001 suggesting that data were appropriate for factor analysis. Exploratory factor analysis (EFA) was conducted on a random selection (*n* = 400 responders). Confirmatory factor analysis (CFA), to test the model from the EFA, was conducted on the remaining material (*n* = 539 responders). Around 75% of the responders had answered all questions. When looking at non-response on specific items, the question “How large of a problem do you think lack of knowledge is for medication errors?” had the highest partial non-response of around 9%. The other items had a partial non-response between 0% and 5%. No systematic differences in non-response were found when stratifying on sex, age groups or profession.

The EFAs were fitted with different numbers of factors using a polychoric correlation matrix. The choice of number of factors for the EFA-model was based mainly on total variance explained by each factor solution [16]. A parallel analysis was also performed to guide in the choice of number of factors [17]. Extraction of factors was done using Principal Axis Factoring (PAF) method [18]. Furthermore, Cronbach’s *α* was examined for all items loading on a factor, with ≥0.7 used as guideline [19]. Choice of number of factors was also guided by how clear/simple the path diagram was and if the grouping of items was judged as clinically meaningful by the researchers. Path diagrams were constructed based on different rotation-techniques. Because extracted factors were mainly correlated, oblique rotations promax, oblimin and obiqmax were used [20]. Level for acceptable factor loading was set at ≥0.3 [21]. Based on the EFA, the residual correlation matrix was 0.048. Overall, based on these criteria, we decided on a model with four factors. The four dimensions were discussed and labelled by the research team.

Analysis of construct validity was done by confirmatory factor analyses (CFA) on a separate subset of the responses. The CFA was estimated using maximum likelihood with Satorra–Bentler scale corrections (MLSB). This method is robust against violations to the multivariate normality assumption [22]. For determining goodness of fit, the following measures were used: standardized root mean square residual (SRMR), cut-off <0.08 indicates acceptable fit; root mean square error of approximation (RMSEA), cut-off <0.06 and upper limit of 90% of confidence interval <0.08 indicate acceptable fit; normed fit index (NFI), cut-off > 0.95 indicates an adequate fit; comparative fit index (CFI), cut-off >0.95 indicates an adequate fit [23].

Internal consistency was assessed by the data of the factor analysis as measure of Cronbach’s α. When examining Cronbach’s *α* for each dimension we excluded the question “The same medication substance can have many different trade names. How big of a risk do you think that is when it comes to medication errors?” since the Cronbach’s *α* was improved by excluding the item. Furthermore, the question only weakly correlated with other items within the dimension.

The patient survey could not be validated due to low response rate. All statistical analyses of the survey data were performed using SAS® (Version 9.4, SAS Institute Inc., Cary, NC).

### Analysis of survey responses

Non-parametric analyses were used concerning areas of risk for medication and diagnostic errors (answers were given on a 11-degree scale from 0 to 10). Data were visualized in box plots and medians reported. Pairwise confidence intervals (Wilcoxon–Mann–Whitney) between the different groups within HCPs (doctors, nurses, practice managers) were calculated to check that they could be reported together as one group, data not shown. To compare the professionals and the patients regarding the questions about continuity and knowledge, Wilcoxon–Mann–Whitney test was used where both raw *p*-value and Bonferroni-adjusted *p*-value were reported. Top ranking solutions were reported descriptively.

## Results

### Construct validity

Factor analysis yielded four dimensions: Patient-provider level; Support systems for every day clinical work; Shared information and cooperation between different caregivers, and; Risks in the environment/in the work conditions. Item clustering is displayed in Table 1 and in a path diagram (Supplementary Appendix 4).

**Table 1.** Dimensions (4) and items (21). Survey to 930 personnel in primary care (850-930 observations).

Question	Dimensions with definitions and items
	**Dimension 1:** Patient-provider level (7 items)
	**Definition:** The knowledge and information required in the meeting (doctor’s own knowledge or knowledge about the patient’s medication received from other health care professionals), the quality of the communication and the degree of understanding each other.
Q13	How large of a problem do you think lack of communication between groups of personnel is for medication errors?
Q12	How large risk do you believe lack of education is for medication errors?
Q28	How large risk do you believe bad consultation technique is for medication errors?
Q15	How large of a problem do you think lack of communication between personnel and patients is for medication errors?
Q34	How large of a problem do you think lack of communication between personnel and patients is for diagnostic errors?
Q30	How large of a problem do you think lack of knowledge is for diagnostic errors?
Q51	How large of a problem do you think lack in language skills in patients or personnel is for diagnostic errors?
	**Dimension 2:** Support systems for every day clinical work (5 items)
	**Definition:** Peer support/Communication with other health care professionals, interprofessional rounds. Incident reporting and feed-back regarding both medication errors and diagnostic errors. Using knowledge support systems in order to establish the correct diagnosis.
Q19	How large of a problem do you think it is to lack routines for incident reporting and feedback for medication errors?
Q40	How large of a problem do you think it is to lack routines for incident reporting and feedback for diagnostic errors?
Q32	How large of a problem do you think lack of communication between groups of personnel is for diagnostic errors?
Q33	How important do you think interprofessional rounds are to prevent diagnostic errors?
Q49	How large of a problem do you think it is with underuse of knowledge support systems for diagnostic errors?
	**Dimension 3:** Shared information and cooperation between different caregivers. (4 items)
	**Definition:** Transfer of care and cooperation between primary and secondary care. The existence of a nationwide online medication record, accessible for primary care, secondary care and the patient.
Q22	How large of a risk for medication errors do you think it is with poor cooperation between secondary and primary care?
Q45	How large of a risk for diagnostic errors do you think it is with poor cooperation between secondary and primary care?
Q21	How large of a risk for medication errors do you think it is that there is not at nationwide online medication record that all stakeholders can access?
Q44	How large of a risk for diagnostic errors do you think it is that there is not at nationwide online medication record that all stakeholders can access?
	**Dimension 4:** Risks in the environment/in the work conditions (5 items)
	**Definition:** Stress and lack of time. High work load. Poor continuity of care.
Q17	How large of a problem do you think stress/lack of time is for medication errors?
Q37	How large of a problem do you think stress/lack of time is for diagnostic errors?
Q18	How large of a problem do you think high work load is for medication errors?
Q26	How large of a risk do you think it is with poor continuity of care when it comes to medication errors?
Q47	How large of a risk do you think it is with poor continuity of care when it comes to diagnostic errors?

The statistical model had an acceptable fit to the observations. The overall measure for the root mean square off-diagonal residuals was 0.048 for the EFA-data, which is below the cut-off 0.05. The fit indices evaluating the overall fit of the confirmatory factor analysis are presented in Supplementary Appendix 4. When comparing the observed estimate to the cut-off levels, two indices (CFI and NFI) where below the cut-off indicating a poor fit. One measure (SRMR) indicated an adequate fit. The value of the RMSEA estimate is above the cut-off but the upper limit of the confidence interval is below the cut-off.

### Internal consistency

All four dimensions had an adequate level of internal consistency in both EFA-data and CFA-data (Cronbach’s *α* above 0.7; Supplementary Appendix 4).

### Response rate and demographics

There were 939 HCPs (581 physicians, 204 nurses and 154 practice managers) and 80 patients that responded (Table 2). Response rates were and 26% for HCPs and 19% for patients. Respondents were from all over the country. Sex, age and profession are reported in Table 2.

**Table 2.** Characteristics of patients and healthcare professionals (*n* = 1 019) who answered the survey regarding patient safety in primary health care during 2018.

	Patients (*N* = 80)		Health care professionals (*N* = 939)					
			Medical doctors		Nurses		Practice managers	
	*N* = 80	Percentage (%)	*N* = 581	Percentage (%)	*N* = 204	Percentage (%)	*N* = 154	Percentage (%)
Sex								
Women	49	61	367	63	196	96	110	71
Men	28	35	204	35	6	2.9	41	27
No answer	3	3.8	10	1.7	2	1.0	3	2.0
Age								
20 years or less	14	18						
21–40 years	27	34	407	70	50	22	11	7.2
41–60 years	25	31	164	28	119	58	104	68
Over 60 years	14	17	7	1.2	33	16	38	25
No answer			3	0.5	3	1.5	1	0.7

### Risk rating

**Figure 1.**
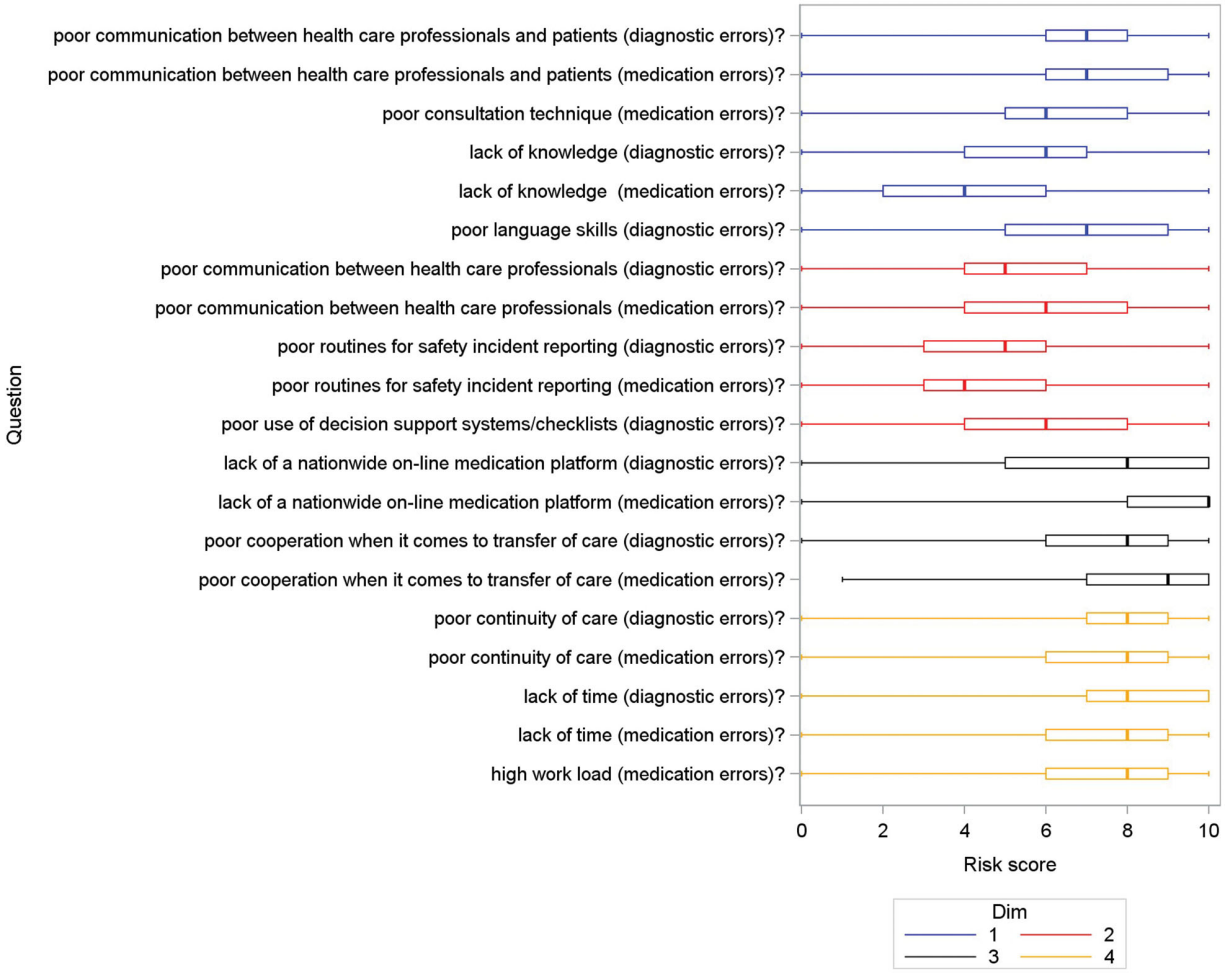
Problems/risks ranked from 0 to 10 by health care professionals during 2018, number of responders 939. Results as box-plots with median as the solid line inside the box, Q1(25th percentile)/Q3(75th percentile) as the left/right edge of the box and whiskers representing minimum and maximum (range). Questions written out in survey, Supplementary Appendix 1.

**Figure 2.**
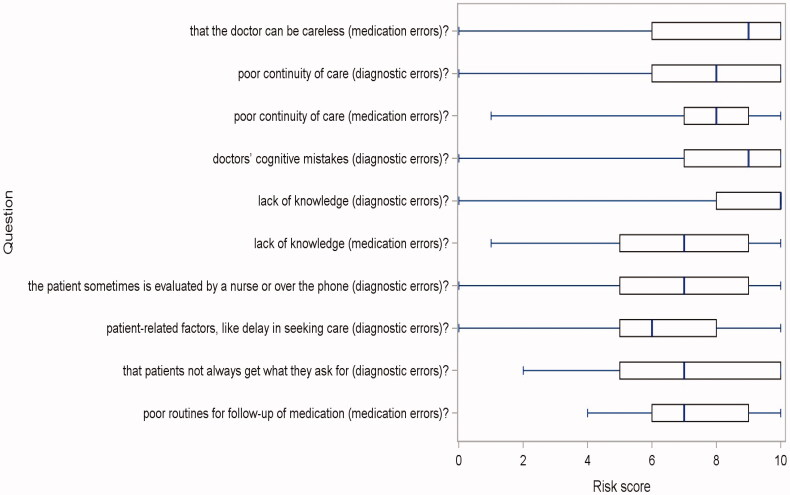
Problems/risks ranked from 0 to 10 by patients during 2018, number of responders 80. Results as box-plots with median as the solid line inside the box, Q1(25th percentile)/Q3(75th percentile) as the left/right edge of the box and whiskers representing minimum and maximum (range). Questions written out in survey, Supplementary Appendix 2.

The risk ratings of HCPs are displayed in Figure 1, divided in the four dimensions from the factor analysis. The risk ratings of patients are displayed in Figure 2. Both HCPs and patients rated relational continuity of care as important for safer care. Perceived professional knowledge was seen as important from the patients’ perspective (median 10 for diagnostic and 7 for medication errors). There was a difference between HCPs and patients regarding the importance of knowledge, Bonferroni-corrected *p*-value < 0.001 (interpreted as a shift in location) that was not seen regarding continuity of care (raw *p*-value around 0.7). This result was found both for medication and diagnostic errors.

### Possible solutions/risk mitigation

Professionals wanted patients to be assigned to their own GP in order to improve continuity of care. Many HCPs wanted improved communication with patients using written individual plans. The solutions with the highest number of top rankings from HCPs are shown in Table 3, alternative solutions are found in Supplementary Appendix 1–3.

**Table 3.** Ways/solutions to mitigate risk of diagnostic and medication errors that were rated as ‘of high importance’ by health care professionals during 2018, number of respondents 939.

	Area of risk	Solutions	% of respondents that ranked this solution highest within each area of risk
Diagnostic errors	Continuity of care	Increase the number of patients that have a personal GP	33
	Transfer of care	Clarify the responsibilities in diagnostics between primary and secondary	37
	Communication between health care professionals and patient	Written information to the patient regarding individual plan for the diagnostic process, test-results and so on	56
	Stress/lack of time	Prioritising of the most severely ill patients	29
	Organizational structure	Clarify the mission of primary care	48
Medication errors	Continuity of care	Increase the number of patients that have a personal GP	39
	Transfer of care	Medication review with the patient and prescription of relevant medications at the hospital before discharge	59
	Communication between health care professionals and patient	Medication review with the patient	50
	Medication labelling	Generic prescription	53
	Communication between groups of professionals	Rounds/meetings on a regular basis	25
	Consultation technique	Training in ‘teach back’ (patient involvement and understanding are secured by the patient explaining what he/she has understood from the consultation)	60

Alternative solutions supplied in Supplementary Appendices 1–3. Solutions from patients are presented only in the text, not in table format.

Patients stressed the importance of nationwide online medication records, a thorough physical examination, and routines for follow-up if tests are normal but symptoms remain, for example information about when and who to contact. Better follow-up routines were also rated of high importance (Supplementary Appendix 3).

## Discussion

### Main findings

The survey to HCPs measured four dimensions: Patient-provider level, Support systems for every day clinical work, Shared information and cooperation between different caregivers and Risks in the environment. Validity as measured by factor analysis was tentatively acceptable. The patient survey could not be validated.

The survey response rates were too low to allow for conclusions. However, answers stressed the importance of relational continuity of care and pointed towards potential differences between professional and patient experience regarding professional knowledge.

### Comparison with previous studies

The factor model identified in the HCP survey reflects the fact that diagnostic and medication errors can have the same underlying explanations. Answers to questions about diagnostic errors and medication errors were, therefore, often grouped together. Similarly, in other studies different types of errors had the same contributing factors such as communication breakdown and situational factors [12].

The four dimensions corresponded to contributing factors from other frameworks, supporting the construct validity of the survey even if the statistics provided conflicting results. The YCFF for patient safety incidents in hospitals has identified the factors active failures, situational factors, local working conditions, latent/organisational factors and latent/external factors [10,24]. The dimension Patient-provider level corresponds to “Active failures” in YCFF, including patient-provider communication [10]. The dimension Support systems corresponds to some extent to “Situational factors” and “Local working conditions” in YCFF. The dimension Transfer of care and cooperation corresponds to some extent to “Latent/organisational factors”. The dimension Risks in the environment/in the work conditions correspond to some aspects of “Local working conditions”.

In another study, five additional factors were suggested as unique for primary care and not included in in YCFF; timely access, primary-secondary interface, continuity of care, task performance (skill and competence) and enough time in the consultation [11]. Primary–secondary interface reflects our dimension Shared information and cooperation and the risk of horizontal task-shifting has been pointed out earlier [25]. Continuity of care reflects our dimension Risks in the environment/in the work conditions. Communication was identified as central in the YCFF, in the primary care study and when exploring the perspectives of GPs [12], as in our survey to HCPs.

Lack of relational continuity of care was rated as an important risk among HCPs and patients in alignment with previous studies [26]. There are other types of continuity of care; informational continuity and management continuity [27], but the questions in our survey were formulated as relational continuity. In addition, poor communication was identified, as shown previously [12]. Stakeholders’ priorities concerning patient safety problems and solutions have received little attention so far, especially regarding patients with experience of harm. Some studies have suggested interesting patient-centred recommendations for improving patient safety including patient-centred communication, timely appointments, active monitoring, teamwork and better work environment [28], but these suggestions did not specifically come from patients that had experienced harm in primary care. In our study, patients who had experienced harm considered lack of physician knowledge as a problem and wanted the doctor to examine them thoroughly, perhaps signalling a lack of trust in the outcome of the consultation.

HCPs and patients in our study ranked risks and solutions similar to a dataset from the UK regarding cooperation between primary and secondary care [29,30]. The current study identifies that a national on-line electronic medication platform, where patients as well as health care could see prescribed medications, could strengthen informational continuity.

### Strengths and limitations

The study has several limitations in survey validation. We used a subset sample drawn only from the completed surveys to HCPs with all items answered for validation of psychometric properties. Respondents with shorter experience of healthcare, older respondents and doctors were more likely to turn in completed surveys, but 75% of the respondents answered all the questions of in the survey. The age distribution among patients show fewer respondents in the group over 60 years of age which might reflect the rather complex survey. Responders may leave questions unanswered if the item is not relevant to them, possibly explaining why doctors answered all questions to a greater extent than nurses and managers. This can result in ascertainment bias where all members of the target population are not equally represented. The patient survey was not subject to factor analysis because it had fewer respondents; however, 40% of the questions were built in the same manner as in the survey to HCPs.

Several respondent characteristics may have led to bias. GP practices were randomly selected, but among responders the persons with interest in patient safety might be overrepresented. Additionally, we excluded cases in which error had resulted in patient death as we did not want to risk evoking pain in relatives, which could also lead to biased responses.

The HCP survey addressed 22 risks, too many to enable reliable conclusions in comparison of ranking. The median varied, however, from low (4) to high (10), indicating which risks were considered most important to address.

The low response rate (26% for HCPs and 19% for patients) limits conclusions from the study. Women are in majority among HCPs reflecting the demographics of GPs and nurses in primary health care in Sweden. More patients were women than men, consistent with the fact that women report harm more often [31].

The surveys used in this study were constructed using a qualitative analysis of HCPs’ understanding of safety risks and ways to mitigate those risks in PHC, increasing the surveys’ relevance [13]. Another strength of the study was that it included patients that had experienced preventable harm in the form of diagnostic error or medication error in primary health care giving their perspective on risks and solutions. Earlier studies have asked patients in general, not patients that have been subject to harm [28,32].

### Clinical implications

The HCP survey shows somewhat conflicting results regarding model fit. The answers indicate that patient and HCP perspectives complement each other. Although our findings cannot be used for policy or practice changes due to low response rate, the HCP survey can be used in future validations and could guide research and in extension improvement of care. There are practically no instruments for investigating patient safety in primary care. At present, Manchester Patient Safety Framework (MaPSaF) is the only tool available and it focuses on patient safety culture, not potential interventions. Our surveys are action-oriented and aim to explore potential points of intervention to improve patient safety.

### Implications for future research

Certain areas of risks and solutions received high ratings, indicating areas of future interest. The surveys should be adjusted and simplified according to the dimensions and then replicated with the aim to obtain a higher response rate for further validation and more reliable results, maybe with patients matched with professionals.

Patients perceived a lack of GP knowledge not recognised by HCPs, a difference that could be further explored in order to understand if there is a potential lack of knowledge or if this perception could be a result of poor communication.

## Conclusion

The HCP survey had to some contradicting results regarding model fit and may be tentatively acceptable but its validity needs further study. The survey might benefit from being shortened and simplified according to found dimensions. Survey answers from HCPs indicate a high value of relational continuity of care, the importance of a nationwide on-line medical platform and the crucial cooperation needed in transfer of care.

## Supplementary Material

Supplemental Material

Supplemental Material

Supplemental Material

Supplemental Material

## Data Availability

Datasets used and/or analysed during the current study are available from the corresponding author on reasonable request.
