# Supplementary material for: Validation and initial results of surveys exploring perspectives on risks and solutions for diagnostic and medication errors in primary care in Sweden
Source: Scand J Prim Health Care. 2020 Dec 11;38(4):381–90. doi: 10.1080/02813432.2020.1841531 (PMC7782021; doi:10.1080/02813432.2020.1841531)
Supplement: Supplemental Material [file IPRI_A_1841531_SM8944.docx]

***Appendix 4*** ***Results of confirmatory factor analysis***

Path diagram for the CFA


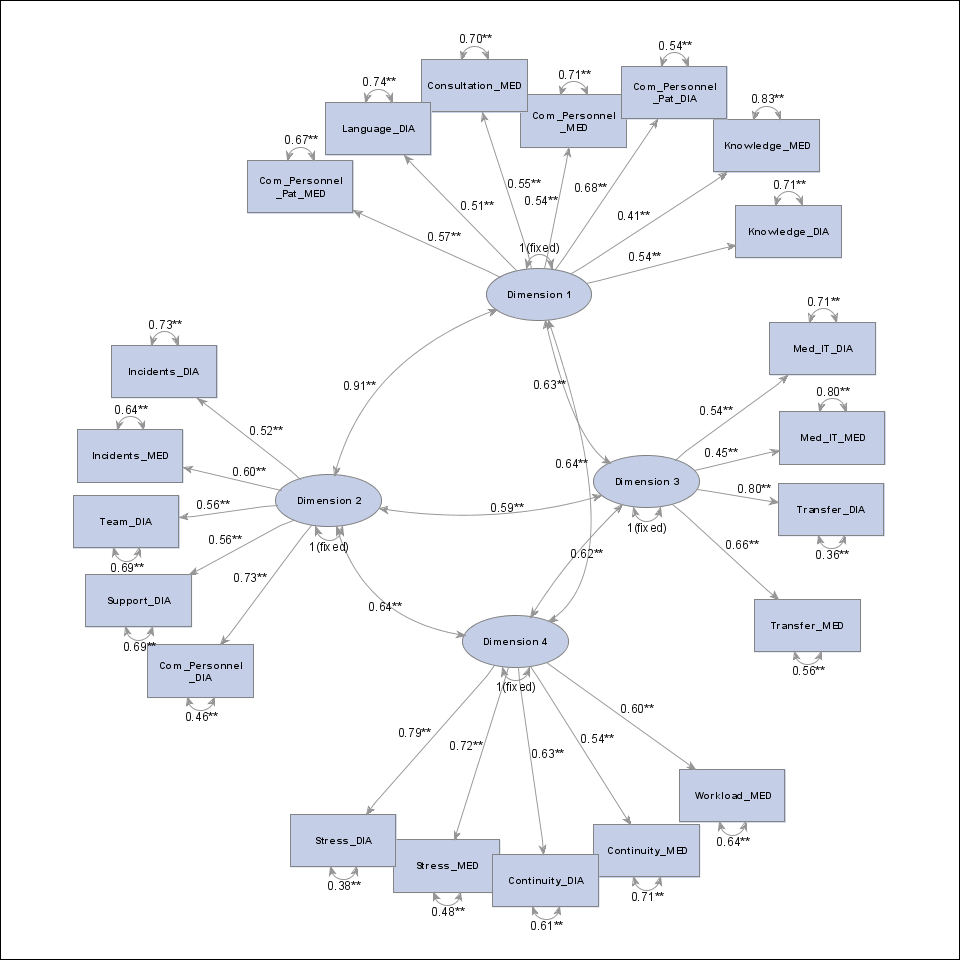


**= Estimate is significant at significance level 1%.

Dimensions (4) with definitions and items/questions (21). Survey to 930 personnel in primary care (850-930 observations)

| **1** | **Question abbreviation** | **Dimension 1: Patient-provider level (7 items)** |
| --- | --- | --- |
|  |  | **Definition: The knowledge and information required in the meeting (doctor’s own knowledge or knowledge about the patient’s medication received from other health care professionals), the quality of the communication and the degree of understanding each other.** |
| Q13 | Com_personnel_MED | How large of a problem do you think lack of communication between groups of personnel is for medication errors? |
| Q12 | Knowledge_MED | How large risk do you believe lack of education is for medication errors? |
| Q28 | Consultation_MED | How large risk do you believe bad consultation technique is for medication errors? |
| Q15 | Com_Personnel_Pt_MED | How large of a problem do you think lack of communication between personnel and patients is for medication errors? |
| Q34 | Com_Personnel_Pt_DIA | How large of a problem do you think lack of communication between personnel and patients is for diagnostic errors? |
| Q30 | Knowledge_DIA | How large of a problem do you think lack of knowledge is for diagnostic errors? |
| Q51 | Language_DIA | How large of a problem do you think lack in language skills in patients or personnel is for diagnostic errors? |
| **2** |  | **Dimension 2: Support systems for every day clinical work (5 items)** |
|  |  | **Definition: Peer support/Communication with other health care professionals, interprofessional rounds. Incident reporting and feed-back regarding both medication errors and diagnostic errors. Using knowledge support systems in order to establish the correct diagnosis.** |
| Q19 | Incidents_MED | How large of a problem do you think it is to lack routines for incident reporting and feedback for medication errors? |
| Q40 | Incidents_DIA | How large of a problem do you think it is to lack routines for incident reporting and feedback for diagnostic errors? |
| Q32 | Com_personnel_DIA | How large of a problem do you think lack of communication between groups of personnel is for diagnostic errors? |
| Q33 | Team_DIA | How important do you think interprofessional rounds are to prevent diagnostic errors? |
| Q49 | Support_DIA | How large of a problem do you think it is with underuse of knowledge support systems for diagnostic errors? |
| **3** |  | **Dimension 3: Shared information and cooperation between different caregivers. (4 items)** |
|  |  | **Definition: Transfer of care and cooperation between primary and secondary care. The existence of a nationwide online medication record, accessible for primary care, secondary care and the patient.** |
| Q22 | Transfer_MED | How large of a risk for medication errors do you think it is with poor cooperation between secondary and primary care? |
| Q45 | Transfer_DIA | How large of a risk for diagnostic errors do you think it is with poor cooperation between secondary and primary care? |
| Q21 | Med_IT_MED | How large of a risk for medication errors do you think it is that there is not at nationwide online medication record that all stakeholders can access? |
| Q44 | Med_IT_DIA | How large of a risk for diagnostic errors do you think it is that there is not at nationwide online medication record that all stakeholders can access? |
| **4** |  | **Dimension 4: Risks in the environment/in the work conditions (5 items)** |
|  |  | **Definition: Stress and lack of time. High work load. Poor continuity of care.** |
| Q17 | Stress_MED | How large of a problem do you think stress/lack of time is for medication errors? |
| Q37 | Stress_DIA | How large of a problem do you think stress/lack of time is for diagnostic errors? |
| Q18 | Workload_MED | How large of a problem do you think high work load is for medication errors? |
| Q26 | Continuity_MED | How large of a risk do you think it is with poor continuity of care when it comes to medication errors? |
| Q47 | Continuity_DIA | How large of a risk do you think it is with poor continuity of care when it comes to diagnostic errors? |

Internal consistency by Cronbach’s alpha

|  | **Dimension** | **EFA-data** | **CFA-data** |
| --- | --- | --- | --- |
| 1 | Patient provider level | 0.745 | 0.731 |
| 2 | Support systems for every day clinical work | 0.756 | 0.795 |
| 3 | Shared information and cooperation between different caregivers. | 0.765 | 0.753 |
| 4 | Risks in the environment/in the work conditions | 0.720 | 0.754 |

Summary of fit indexes

| **Fit index** | **Fit statistic** | **Cut-off adequate fit *** |
| --- | --- | --- |
| Standardized root mean square residual (SRMR) | 0.066 | <0.08 |
| Root mean square error of approximation (RMSEA) | 0.072 (0.065;0.078) | <0.06 & 90 % upper limit < 0.08 |
| Normed Fit Index (NFI) | 0.755 | >= 0.95 |
| Comparative fit index (CFI) | 0.807 | >= 0.95 |

*Schreiber, James & Nora, Amaury & Stage, Frances & Barlow, Elizabeth & King, Jamie. (2006). Reporting Structural Equation Modelling and Confirmatory Factor
