# Supplementary material for: Validation and initial results of surveys exploring perspectives on risks and solutions for diagnostic and medication errors in primary care in Sweden
Source: Scand J Prim Health Care. 2020 Dec 11;38(4):381–90. doi: 10.1080/02813432.2020.1841531 (PMC7782021; doi:10.1080/02813432.2020.1841531)
Supplement: Supplemental Material [file IPRI_A_1841531_SM8894.docx]

***Appendix 3 Details on ranking of different solutions***

Possible solutions or ways to mitigate risk, perspective of patients. Percentage (%) that ranked each solution as the most important.

**Diagnostic errors**

**Medication errors**

**Possible solutions or ways to mitigate risk, perspective of health care professionals. Percentage (%) that ranked each solution as the most important.**

**Diagnostic errors**

**Medication errors**
