# Supplementary material for: Validation and initial results of surveys exploring perspectives on risks and solutions for diagnostic and medication errors in primary care in Sweden
Source: Scand J Prim Health Care. 2020 Dec 11;38(4):381–90. doi: 10.1080/02813432.2020.1841531 (PMC7782021; doi:10.1080/02813432.2020.1841531)
Supplement: Supplemental Material [file IPRI_A_1841531_SM8870.pdf]

For a safer primary health care. Survey to physicians, nurses and practice managers.

The survey deals with MEDICATION errors and DIAGNOSTIC errors in primary health care and takes 7-10 minutes to complete.

The questions and suggested solutions are based on the views of physicians, nurses and practice managers in primary health care.

1) Do you consent to participate in this patient safety study according to the supplemented information? Your answers are anonymised.

- ○ Yes (otherwise do not complete the survey)

2) What profession do you have?

- ○ Physician
- ○ Nurse
- ○ Other, specify:

3) Are you practice manager?

- ○ Yes
- ○ No

4) In what region do you work?

- ○ Stockholms läns landsting
- ○ Region Uppsala
- ○ Landstinget Sörmland
- ○ Region Östergötland
- ○ Region Jönköpings län
- ○ Region Kronoberg
- ○ Landstinget i Kalmar län
- ○ Region Gotland
- ○ Landstinget Blekinge
- ○ Region Skåne
- ○ Region Halland
- ○ Västra Götalandsregionen
- ○ Landstinget i Värmland
- ○ Region Örebro län
- ○ Region Västmanland
- ○ Landstinget Dalarna
- ○ Region Gävleborg
- ○ Region Västernorrland
- ○ Region Jämtland Härjedalen
- ○ Västerbottens läns landsting
- ○ Region Norrbotten

5) How many patients are listed at your GP practice?

- ○ Fewer than 5000
- ○ 5000-10000
- ○ 10000-15000
- ○ 15000-20000
- ○ More than 20000

6) How far is it from your GP practice to the nearest hospital?

- ○ Under 50 kilometers
- ○ Over 50 kilometers

7) Do you work at a private or public GP practice?

- ○ Private
- ○ Public

8) Your sex

- ○ Female
- ○ Male
- ○ Do not want to specify

9) Your age

- ○ 20-30 years
- ○ 31-40 years
- ○ 41-50 years
- ○ 51-60 years
- ○ 61-70 years
- ○ older than 70 years

10) How many years have you worked in your profession?

- ○ 0-5 years
- ○ 5-10 years
- ○ 11-20 years
- ○ 21-30 years
- ○ 31-40 years
- ○ 41-50 years
- ○ more than 50 years

The first part of the survey is about medication errors, that is, errors in prescribing, acquiring, storing, dispensing, or administering medication with the result that the patient fails to receive the correct drug or the indicated proper drug dosage.

11) How big of a of a problem do you think lack of knowledge is for medication errors?

- 0 not a problem at all
- 1
- 2
- 3
- 4
- 5
- 6
- 7
- 8
- 9
- 10 a very big problem

12) How important do you think further training is to reduce the risk for medication errors?

- 0 not important at all
- 1
- 2
- 3
- 4
- 5
- 6
- 7
- 8
- 9
- 10 very important

13) How big of a of a problem do you think poor communication between groups of health care professionals is, regarding medication errors?

- ○ 0 not a problem at all
- ○ 1
- ○ 2
- ○ 3
- ○ 4
- ○ 5
- ○ 6
- ○ 7
- ○ 8
- ○ 9
- ○ 10 a very big problem

The solutions that you are asked to rank have their origin in our previous questionnaire about patient safety. You can add your own solutions at the end of the survey.

14) How do you rank these suggestions of solutions regarding communication between groups of professionals, when it comes to medication errors?

	1 Least important suggestion	2	3	4 Most important suggestion
Rounds/meetings on a regular basis	○	○	○	○
Proximity between doctors and nurses	○	○	○	○
Both doctor and nurse participate in the patient consultation	○	○	○	○
Guidelines regarding division of responsibilities between doctors and nurses in primary health care	○	○	○	○

15) How big of a of a risk do you think poor communication between primary care providers (physicians and nurses) and patients is for medication errors?

- 0 no risk
- 1
- 2
- 3
- 4
- 5
- 6
- 7
- 8
- 9
- 10 a very big risk

16) How do you rank these suggestions of solutions, when it comes to medication errors?

	1	2	3
Written information to patients about possible side effects of medication	○	○	○
An up to date list of medication, on paper or on-line	○	○	○
Medication review with the patient	○	○	○

17) How big of a of a problem do you think lack of time/stress is for medication errors?

- 0 not a problem at all
- 1
- 2
- 3
- 4
- 5
- 6
- 7
- 8
- 9
- 10 a very big problem

18) How much do you think a reduced work load in primary health care would decrease the risk for medication errors?

- 0 to a very low degree
- 1
- 2
- 3
- 4
- 5
- 6
- 7
- 8
- 9
- 10 it is of upmost importance to reduce the work load to decrease the risk of error

19) How big of a of a problem do you think that poor routines for safety incident reporting is for medication errors?

- 0 not a problem at all
- 1
- 2
- 3
- 4
- 5
- 6
- 7
- 8
- 9
- 10 a very big problem

20) How do you rank these solutions regarding incident reporting when it comes to medication?

	1	2	3
Supportive climate when it comes to incident reporting			
Arenas for discussion and learning regarding incidents that have taken place			
Good routines for handling/follow-up of incidents (for example digital system for reporting and yearly/quarterly reports)			

21) How important do you think a nationwide electronic medication platform for all stakeholders (GP practice, hospital, pharmacy, patient) would be to decrease the risk for medication errors?

- 0 not important at all
- 1
- 2
- 3
- 4
- 5
- 6
- 7
- 8
- 9
- 10 very important

22) How big of a of a risk do you think that poor cooperation regarding transfer of care is, when it comes to medication errors?

- 0 minimal risk
- 1
- 2
- 3
- 4
- 5
- 6
- 7
- 8
- 9
- 10 very big risk

23) How do you rank these solutions regarding transfer of care when it comes to medication errors?

	1	2	3	4
Clarify the responsibility for prescribing medication between different specialties	○	○	○	○
Medication review with the patient and prescription of relevant medications at the hospital before discharge	○	○	○	○
Increased use of digital support for discharge that are shared between primary and secondary care	○	○	○	○
Increased knowledge in the hospital-based care about digital prescribing using ‘individual dosage delivery’ of medication	○	○	○	○

24) The same medication substance can have many different tradenames. How big of a of a risk do you think that is when it comes to medication errors?

○ 0 no risk
○ 1
○ 2
○ 3
○ 4
○ 5
○ 6
○ 7
○ 8
○ 9
○ 10 a very big risk

25) How do you rank these suggestions of solutions?

	1	2
Generic prescription	○	○
Better labelling of medication, so it is clear what medication has been prescribed.	○	○

26) How big of a of a problem do you think that poor continuity of care (that the patient can meet different health care professionals every visit) is for the risk of medication errors?

- ○ 0 not a problem at all
- ○ 1
- ○ 2
- ○ 3
- ○ 4
- ○ 5
- ○ 6
- ○ 7
- ○ 8
- ○ 9
- ○ 10 a very big problem

27) How do you rank these suggestions of solutions when it comes to the risk for medication errors?

	1	2	3	4
Increase the number of patients that have a personal GP	○	○	○	○
Increase the number of patients that are listed to a nurse	○	○	○	○
Schedule the next visit when at the GP practice	○	○	○	○
Work in teams	○	○	○	○

28) How big of a of a problem do you think that poor consultation technique (poor communication between the health care professionals and the patient) is for the risk of medication errors?

- 0 not a problem at all
- 1
- 2
- 3
- 4
- 5
- 6
- 7
- 8
- 9
- 10 very big problem

29) Here we ask you to rank these suggestions of solutions when it comes to the risk for medication errors.

	1	2
Education/further training in consultation techniques for health care professionals (ideas, concerns, expectations)	○	○
Training in 'teach back' (patient involvement and understanding are secured by the patient explaining what he/she has understood from the consultation)	○	○

The second part of the survey is about DIAGNOSTIC errors, that is errors in the diagnostic process that lead to the patient not receiving the diagnosis in a timely manner to receive relevant treatment. For example, a diagnostic error can be cancer that has time to metastasis, a myocardial infarction that is misinterpreted, or a fracture that is not x-rayed.

30) How big of a of a problem do you think that lack of knowledge is for diagnostic errors?

- 0 no problem at all
- 1
- 2
- 3
- 4
- 5
- 6
- 7
- 8
- 9
- 10 a very big problem

Recertification *of health care professionals means a formalised test of specialist competence with fixed time intervals.*

31) How do you rank these suggestions of solutions when it comes to the risk for diagnostic errors?

	1 Least important	2	3	4	5 most important
Recertification for health care professionals					
Further training in patient safety					
Further training in clinical skills (for example CPR)					

	1 Least important	2	3	4	5 most important
Further training in consultation technique	○	○	○	○	○
Discussion of cases with colleges	○	○	○	○	○

32) How big of a of a problem do you think that poor communication between groups of health care professionals is for diagnostic errors in primary health care?

- ○ 0 not a problem at all
- ○ 1
- ○ 2
- ○ 3
- ○ 4
- ○ 5
- ○ 6
- ○ 7
- ○ 8
- ○ 9
- ○ 10 a very big problem

33) How important do you think that rounds in primary health care (with two or more professions) are to avoid DIAGNOSTIC errors?

- ○ 0 not important at all
- ○ 1
- ○ 2
- ○ 3
- ○ 4
- ○ 5
- ○ 6
- ○ 7
- ○ 8
- ○ 9
- ○ 10 very important

34) How big of a of a problem do you think that poor communication between health care professionals and patient is for DIAGNOSTIC errors?

- 0 no risk at all
- 1
- 2
- 3
- 4
- 5
- 6
- 7
- 8
- 9
- 10 very big risk

35) Here we ask you to rank these suggestions of solutions when it comes to the risk for DIAGNOSTIC errors.

	1	2	3	4
Give responsibility to the patient (for example, ask for test results, self-care at home, answer questions before visit for preparation, book new appointment themselves)	○	○	○	○
Written information to the patient regarding individual plan for the diagnostic process, test-results and so on.	○	○	○	○
Pamphlets with information about diseases e.t.c.	○	○	○	○
Information via public channels (TV, newspapers, web pages, social media)	○	○	○	○

36) How big of a of a risk do you think that low degree of patient involvement is for DIAGNOSTIC errors?

- 0 no risk at all
- 1
- 2
- 3
- 4
- 5
- 6
- 7
- 8
- 9
- 10 a very big risk

37) How big of a of a problem do you think that lack of time/stress is for DIAGNOSTIC errors?

- 0 not a problem at all
- 1
- 2
- 3
- 4
- 5
- 6
- 7
- 8
- 9
- 10 very big problem

38) Here we ask you to rank these solutions regarding stress/lack of time when it comes to the risk for DIAGNOSTIC errors.

	1	2	3	4
Longer time per visit	◯	◯	◯	◯
Let other professions than GPs write attestations/sick-leave (physical therapists/occupational therapist)	◯	◯	◯	◯
Prioritising of the most severely ill patients	◯	◯	◯	◯
Time earmarked to take care of test results and follow-up	◯	◯	◯	◯

39) Here we ask you to rank these suggestions of solutions regarding primary care's organisational structure.

	1	2	3
Clarify the mission of primary care	◯	◯	◯
Set a maximum of patients per GP	◯	◯	◯
Renumurate not only visits, but also quality improvement and work with patient safety	◯	◯	◯

40) How big of a of a problem do you think poor routines of writing and follow-up of incident reports are for DIAGNOSTIC errors?

- ◯ 0 not a problem at all
- ◯ 1
- ◯ 2
- ◯ 3
- ◯ 4
- ◯ 5
- ◯ 6
- ◯ 7
- ◯ 8
- ◯ 9
- ◯ 10 a very big problem

41) Here we ask you to rank these solutions regarding patient safety in the area of DIAGNOSTICS.

	1	2	3	4
Ensure feedback to involved persons	○	○	○	○
Supportive climate when it comes to incident reporting	○	○	○	○
Arenas for discussion regarding incidents that have taken place	○	○	○	○
Good routines for handling/follow-up of incidents (for example digital system for reporting and yearly/quarterly reports)	○	○	○	○

42) Which incident reporting system do you have at your GP practice?

- ○ HändelseVis
- ○ FOKUS
- ○ AvIC
- ○ MedControlPro
- ○ Centuri
- ○ Flexite Aha
- ○ Flexsite
- ○ Platina
- ○ Synergi
- ○ LISA
- ○ Manual reporting
- ○ Other
- ○ Do not know

43) Who is responsible for follow-up of incident reports at your workplace? (position, not person)

- ○ Practice manager
- ○ Person assigned at the workplace
- ○ Management
- ○ Other, specify
- ○ Do not know

44) How important do you think that a nationwide on-line medication platform would be to reduce the risk for DIAGNOSTIC errors?

- ○ 0 Not important at all
- ○ 1
- ○ 2
- ○ 3
- ○ 4
- ○ 5
- ○ 6
- ○ 7
- ○ 8
- ○ 9
- ○ 10 very important

45) How big of a of a problem do you think that poor cooperation with hospital-based care regarding transfer of care is for the risk of DIAGNOSTIC errors?

- ○ 0 not important at all
- ○ 1
- ○ 2
- ○ 3
- ○ 4
- ○ 5
- ○ 6
- ○ 7
- ○ 8
- ○ 9
- ○ 10 very important

46) Here we ask you to rank the following suggestions of solutions regarding transfer of care when it comes to DIAGNOSTIC errors.

	1	2	3
Clarify the responsibilities in diagnostics between primary and secondary care (who does what)	○	○	○
Better direct communication with secondary care (via phone, not voicemail)	○	○	○
Change of routine so that specialised care does not return referrals from primary care but instead send them to another instance of specialised care.	○	○	○

47) How big of a of a problem do you think that poor continuity of care is for the risk of DIAGNOSTIC errors?

○ 0 not important at all
○ 1
○ 2
○ 3
○ 4
○ 5
○ 6
○ 7
○ 8
○ 9
○ 10 a very big problem

48) Here we ask you to rank these suggestions of solutions when it comes to the risk for DIAGNOSTIC errors.

	1	2	3	4	5	6
Increase the number of patients that have a personal GP	○	○	○	○	○	○
Increase the number of patients that are listed to a nurse	○	○	○	○	○	○
Schedule the next visit when at the GP practice	○	○	○	○	○	○
Fewer locum physicians	○	○	○	○	○	○
Maximum of patients per GP (for example 1500 patients per doctor)	○	○	○	○	○	○
More ways to communicate with patients (e-mail, video)	○	○	○	○	○	○

49) How big of a of a problem do you think that poor use of decision support systems/checklists/routines is for the risk of DIAGNOSTIC errors?

○ 0 not a problem at all
○ 1
○ 2
○ 3
○ 4
○ 5
○ 6
○ 7
○ 8
○ 9
○ 10 a very big problem

50) Here we ask you to rank these suggestions of solutions regarding decision support to mitigate the risk for DIAGNOSTIC errors.

	1	2	3	4
Support to increased use of standardised course of care for cancer	◯	◯	◯	◯
Support to increased use of the nationwide decision support webpage	◯	◯	◯	◯
Further training in lateral diagnostic thinking (to think of several different possible diagnoses early on)	◯	◯	◯	◯
Good routines for follow-up and for checking on test results when a colleague is absent	◯	◯	◯	◯

51) How big of a of a problem do you think health care professionals' or patients' poor language skills are for the risk of DIAGNOSTIC errors?

◯ 0 not important at all
◯ 1
◯ 2
◯ 3
◯ 4
◯ 5
◯ 6
◯ 7
◯ 8
◯ 9
◯ 10 a very big problem

52) Do you have any more thoughts regarding patient safety in Swedish primary health care? Write here (voluntary)

Thank you for contributing to a safer care!!
